# A Cytological Study of Oral Human Papillomavirus (HPV) Infection Among Electronic Cigarette Smokers in Al-Madinah Al-Munawara

**DOI:** 10.7759/cureus.40421

**Published:** 2023-06-14

**Authors:** Faris M Elmahdi, Razan S Aljohani, Nouf A Alharbi, Sama E Yousef, Noor M Alharbi, Reema B Afasha, Rawan B Aljohani, Yara K Alhejaili, Nasser O Almuzaini

**Affiliations:** 1 College of Medicine, Al-Rayan Colleges, Madinah, SAU; 2 Internal Medicine, Meqat General Hospital, Madinah, SAU

**Keywords:** al-madinah, oral cancer, electronic cigarette, hpv, cytological atypia

## Abstract

Objective

This study aims to determine how electronic cigarette (e-cigarette) use contributes to the spread of the human papillomavirus (HPV) and to assess the potential cellular proliferative activity it may produce.

Methodology

In Madinah, a case-control study was conducted between October 2022 and March 2023. Two oral cytologic smear samples were collected from each of the 500 volunteers; 250 consumers of electronic cigarettes and 250 non-smokers each provided two samples. To detect cytological changes and HPV infection, these samples were stained using Papanicolaou and immunocytochemical techniques.

Results

Twelve (4.8%) of the electronic cigarettes exhibited cytological atypia, while only one (0.4%) of the nonsmokers' group did. Infection with the human papillomavirus (HPV) was detected in eight (3.2%) of the e-cigarette users, but it was only found in two (0.8%) of the nonsmokers (P ≤ 0.05).

Conclusion

Electronic cigarette smoking increases the likelihood of contracting HPV and developing cytological atypia, both of which, if left untreated, can contribute to the development of precancerous and cancerous lesions in the mouth.

## Introduction

Oral infection with the human papillomavirus (HPV) significantly raises the risk of head and neck squamous cell carcinoma (HNSCC), particularly oropharyngeal squamous cell carcinoma [1]. According to the World Health Organization, approximately 6.5 million Saudi Arabian women older than 15 are at risk for developing cervical cancer. An estimated 55 women perish annually in Saudi Arabia from HPV-associated cancers. In Saudi Arabia, HPV-16, HPV-18, and HPV-45 are the most prevalent genotypes and account for approximately 70% of cervical cancer cases [2]. HPV is a minor DNA virus that typically infects the pharynx and skin keratinocytes. So far, at least 200 varieties of HPV have been identified [3]. It was previously believed that HPV caused more than 99 percent of cervical cancer cases worldwide. On the basis of their potential to cause cancer, HPV subtypes have been divided into two categories: high-risk (HR-HPV) and low-risk. HR-HPV genotypes are believed to be the leading cause of cervical cancer. According to the International Agency for Cancer Research (IARC), HR-HPV consists of HPV strains 16, 18, 31, 33, 35, 39, 45, 51, 52, 56, 58, 59, and 66. Low-risk human papillomavirus strains 6, 11, 40, 42, 43, 44, 53, 54, 61, 72, and 81 are classified as LR-HPV [4]. The skin, the pharynx, the tracheobronchial mucosa, the nasal cavity, the paranasal sinus, the esophagus, and the mouth have all been found to harbor HPV infections [5].

The prevalence of HPV-positive oral squamous cell carcinoma (OSCC), which is primarily due to HPV 16, has increased rapidly in younger adults from developed countries like the United States, Australia, and a few European countries [6]. Despite the lack of a systematic analysis of the incidence trend of HPV-positive OSCC in China, HPV infection was recently detected in 17% of OSCCs in China [7], compared to 18% to 35% in the preponderance of studies from developed nations [8]. Molecularly, clinicopathologically, and prognostically, OSCC caused by HPV is distinct from OSCC caused by smoking and imbibing [9].

Given the growing importance of oral HPV infection in oral squamous cell carcinoma (OSCC), it is essential for public health to understand its prevalence among cancer-free individuals and the associated risk factors. Previous estimates of oral HPV infection in normal oral mucosa ranged between 0% and 70% [10].

Both cigarette smoking and electronic cigarette use are associated with an increased prevalence of oral HPV-16 infection [11]. Cigarettes with conventional filters have local and systemic immunosuppressive effects and may irritate the upper aerodigestive tract, thereby facilitating HPV's access to basal cells and infection. Electronic cigarettes can irritate the pharynx and esophagus similarly [12]. E-cigarettes have exploded in popularity among young adults and, if the current trend persists, will eventually surpass traditional cigarettes in terms of utilization. It is unknown, however, whether the use of electronic cigarettes increases the risk of oral HPV infection. 

## Materials and methods

Five-hundred healthy participants were randomly chosen for this cross-sectional study, which was conducted between October 2022 and March 2023. Male Saudi nationals living in Madinah were all participants in this experiment. 300 of the study's 500 participants were determined to be current flavored-e-cigarette smokers, while 200 did not smoke. Those who had ever smoked on a regular basis for 6 months or more by the time of enrollment were categorized as ever smokers; otherwise, they were categorized as never smokers. The intensity and duration of cigarette smoking were dichotomized according to the self-reported age of smoking initiation (≥ 20 years) and the number of cigarettes per day (≥ 20). Both smokers and nonsmokers seemed to be in great health. Any individual with a chronic illness was excluded. Additionally, any volunteers who were located beyond the Al-Madinah Al-Munawara region in Saudi Arabia were excluded. The study did not include anyone who withheld their consent or refused to take part.

Sample collection

Using a wooden tongue depressor, the oral mucosa, which includes the dorsum of the tongue and both cheeks, was sampled. The cells were then evenly distributed between two pieces of clear glass, and while they were still moist, they were quickly fixed in 95% ethyl alcohol. Buccal smears were sent to the histology department at the Al-Rayan College of Medicine for immunohistochemistry and Papanicolaou (Pap) staining in order to identify HPV infection.

Papanicolaou staining technique

After being fixed in 95% ethyl alcohol for 15 minutes, hydrated in 70% ethyl alcohol for two minutes, rinsed in water for two minutes, stained in Harri's haematoxylin for five minutes, rinsed in water, and then differentiated in 1% acid alcohol for two seconds, a buccal smear was ready for examination. Then it was boiled for 10 minutes in regular kitchen water. Smears were dehydrated in 70% alcohol for 2 minutes, changed to 95% alcohol for 2 minutes, stained for 2 minutes with OG6, rinsed in two changes of 95% alcohol for 2 minutes each, allowed to air dry, then rinsed in xylene and mounted in diesterene dibutyl phthalate xylene (DPX). At 40x, the stained smears could be seen [13].

Immunohistochemistry technique

Phosphate-buffered saline (PBS) was used to rinse the smear three times for 3 minutes each. In order to block endogenous peroxidase from working, each slice was given a 15-minute treatment with 0.3% hydrogen peroxide in methanol. A primary mouse monoclonal HPV antibody was used to incubate the sections for 30 minutes at 37 °C (Shanghai GeneTech Company Limited, Shanghai, China). Sections were exposed to the secondary antibody ChemMate EnVision+/HRP (Dako Denmark A/S, Glostrup, Denmark). for 30 minutes at room temperature after two PBS washes. The last chromogen, diaminobenzidine (DAB), was used for 10 minutes, and after three minutes of distilled water rinsing, the antibody for L1 and L2 was visible. After dehydrating in alcoholic solutions, the sections were cleaned with xylene, counterstained with hematoxylin for three minutes, rinsed for five minutes, and mounted with dibutylphthalate polystyrene xylene (DPX). Epithelial cells with brown cytoplasmic staining have been linked to HPV infection [13].

Cytological assessment

An experienced cytotechnologist initially evaluated the smears for quality and dependability. Reliable and repeatable results were achieved with the help of stringent quality control procedures. Cytological atypia was measured using the parameters laid out by Ahmed et al. [14].

Ethical consent

Before the specimen was obtained, each participant was requested to complete an ethical consent form in writing. The Research Ethical Committee of Al-Rayan Colleges created and approved the informed ethical consent form (HA-03-M-122-025).

Data management

Data obtained from this study were analyzed using a statistical package for social science software (SPSS v. 22). A value of 0.05 will be considered the value of statistical significance for all statistical tests in the present study.

## Results

**Figure 1:**
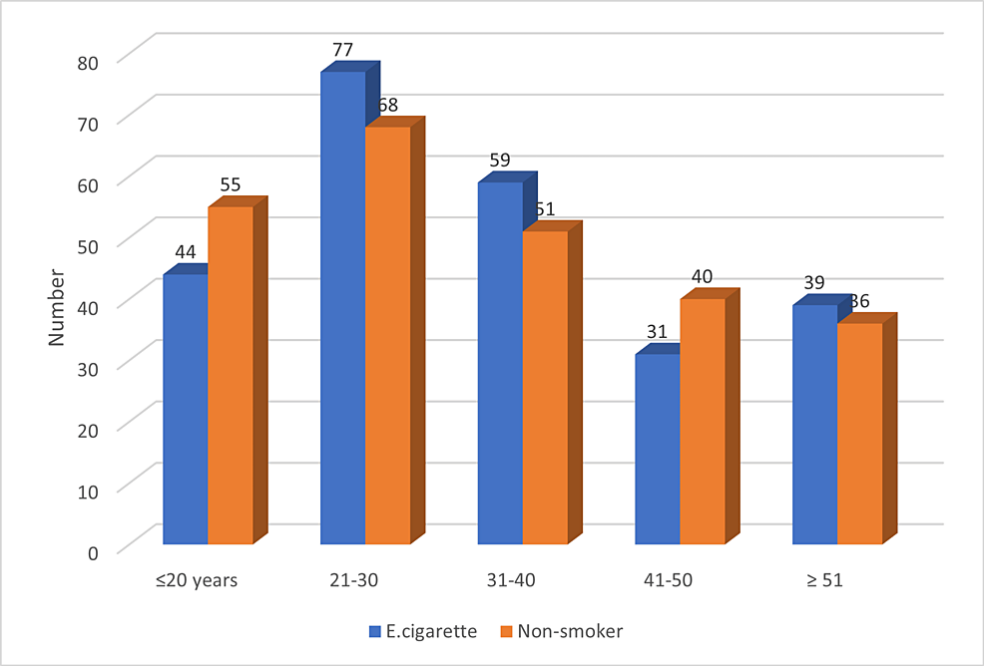
Distribution of study subject by age

In this study, 500 participants - 250 smokers of electronic cigarettes and 250 nonsmokers - with mean ages ranging from 18 to 56 years participated. In general, the distribution of smokers and non-smokers of electronic cigarettes was consistent across all age categories. The age group 20-29 years had the highest concentration of electronic smokers and non-smokers, followed by the 30-39 years age group as indicated in Figure 1.

**Figure 2:**
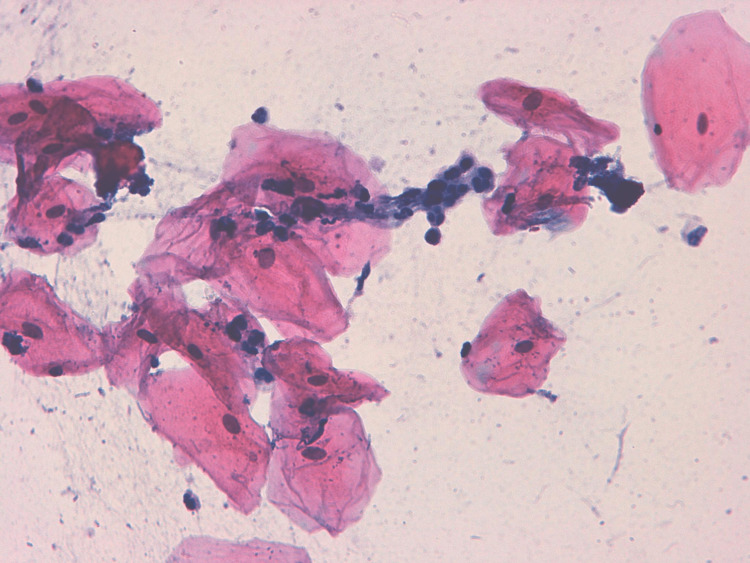
Buccal smear taken from an apparently healthy electronic cigarette smoker - the smear shows inflammatory cells (Pap. stain 40x).

**Figure 3:**
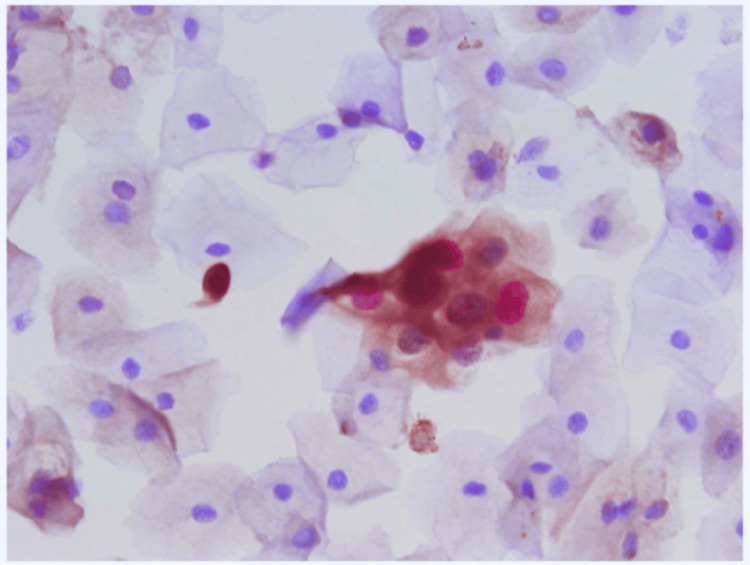
Buccal smear taken from an apparently healthy electronic cigarette smoker - the smear shows extensive L2 expression in the superficial epithelial layers (brown color of the cytoplasm) (immunocytochemistry 40x).

Cytological atypia was found in 12 (4.8%) of the e-cigarette users (OR, 1.12; 95% CI, 1.33-4.48) (P = 0.016) but only in one (0.4%) of the nonsmokers. Inflammatory cell infiltrates were found in 24 (9.6%) of the e-cigarette users' samples (OR, 3.10; 95% CI, 0.32-3.45), but only in five (2%) of the nonsmokers' samples (P = 0.011) as shown in Table 1 and Figure 2. Oral HPV infection was found in eight (3.2%) of the e-cigarette users (OR, 4.84; 95% CI, 1.02-5.12), but it was only found in two (0.8%) of the nonsmokers, as shown in Table 1 and Figure 3. Keratinization was found in six of the e-cigarette users (2.4%) (OR, 7.21; 95% CI, 1.37-6.22) but not in any of the controls.

**Table 1:** Distribution of the study subjects by cytological findings HPV: human papillomavirus

Variable	Electronic Cigarette n (%)	Nonsmoker n (%)	P-value
Atypia	12 (4.8%)	1 (0.4%)	0.016
HPV	8 (3.2%)	2 (0.8)	0.054
Keratinization	6 (2.4%)	0 (0%)	0.142
Inflammation	24 (9.6%)	5 (2%)	0.011

**Figure 4:**
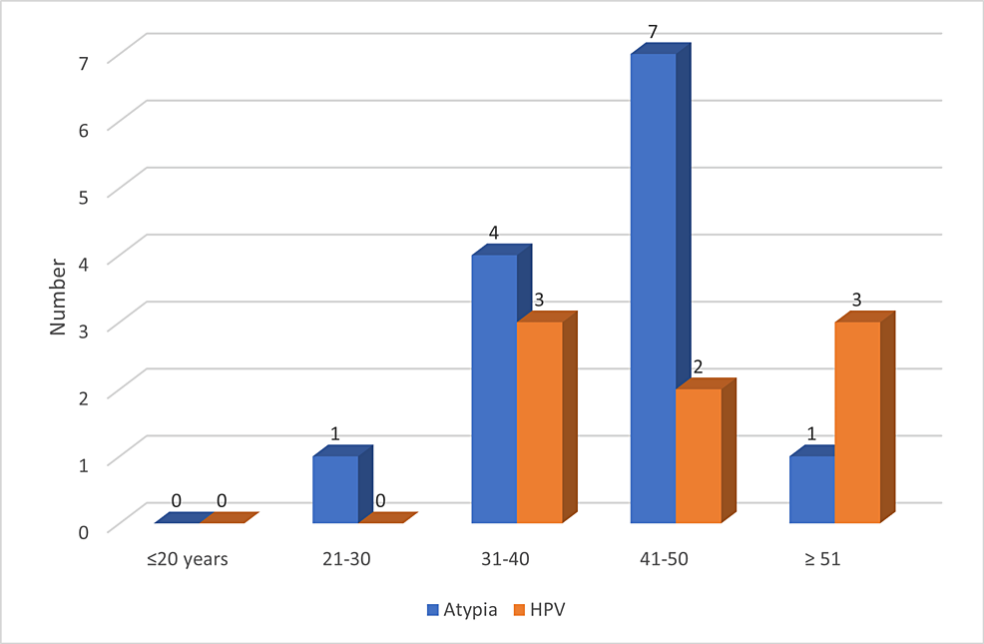
Distribution of the atypia and HPV by age group HPV: human papillomavirus

The preponderance of electronic cigarette smokers with cytological atypia was identified in the age group 41-50 years, followed by 31-40 years, with 7/12 (58.3%) and 4/12 (33.3%), respectively. In addition, the majority of cytological electronic cigarette smokers with viral infections were found in the 31-40 and ≥ 50 age groups, with 3/8 (37.5%), as shown in Figure 4.

With regard to the duration of e-cigarette smokers, the highest atypia cases were found among those who smoked e-cigarettes for a duration of ≥7 years, representing 61.5%, followed by 5-7 years (46.2%). On the other hand, the highest viral infection was detected in the duration of 5-7 years, representing 50%, followed by ≥7 years (40%) 

**Table 2:** Distribution of the cytological findings by duration of e-cigarette smoking. HPV: human papillomavirus

Duration	HPV n (%)	Atypia n (%)	p-value
≤1 year	0 (0%)	0 (0%)	0.32
2-4 years	1 (10%)	0 (0%)	
5-7 years	5 (50%)	6 (46.2%)	
≥7 years	4 (40%)	8 (61.5%)	

## Discussion

Infection with a high-risk HPV strain is a major contributor to the danger of developing mouth cancer. Epithelial surfaces are a common route for HPV to go into the human body. Here, we investigated whether or not regular electronic cigarette users are at increased risk for developing oral precancerous and cancerous lesions, and whether or not oral HPV transmission is possible through such use. The significance of using oral exfoliative cytological procedures for early detection of cytological atypical changes prior to the development of immoral cell lines resulting in oral cancer was also proven by this investigation. All of the participants in the current study were male because of the social stigma associated with smoking.

This population-based investigation discovered a link between oral HPV infection and e-cigarette usage that was statistically significant and unrelated to traditional cigarette smoking. Oral HPV infections and high-risk HPV-16 infections were more likely following dual usage. Although the precise biological mechanisms that can explain these connections are unknown, using e-cigarettes may raise the risk of DNA damage and increase the vulnerability of oral tissues to a high-risk HPV genotype. HPV-16 is the root cause of more than 80% of oropharyngeal cancer cases [15].

Twelve (4.8%) of the smokers exhibited cytological atypia, while only one (0.4%) of the nonsmokers did (P<0.016). Even in the absence of clinical indicators, electronic cigarettes have been linked to the development of oral precancerous lesions, which can progress into oral cancer. In the evolution of malignant progression, cellular changes occur before clinical alterations become evident. [16]. Therefore, identifying and treating high-risk precancerous oral lesions is crucial for reducing oral cancer incidence, prevalence, mortality, and treatment costs. High-risk patients can benefit from oral exfoliative cytology since it can detect changes to the oral mucosa at an early stage [17]. The presence of malignant transformation markers such as atypia, cellular keratinization, hyperchromasia, and higher nuclear/cytoplasmic ratios can be useful in the early detection and diagnosis of oral cancer patients [18,19]. Moreover, in this study, infiltrates of inflammatory cells were more prevalent in cases than in controls, specifically acute inflammatory cells. Regarding age, although the aggregate effects appeared to increase with age, which may be due to prolonged exposure, there were still significant effects on the relatively younger population.

These findings may implicate HPV infection in the pathophysiology of e-cigarette-induced mucosal carcinogenesis. However, long-term epidemiological studies are required to determine the risk associated with e-cigarette use and the development of oral cancer.

Limitation

Study limitations include self-reported smoking, a low sample size, the potential for unmeasured confounding factors, and the low incidence of other HPV subtypes. However, the study's findings are valuable for generating hypotheses for future research on e-cigarette use and oral cancer.

## Conclusions

Electronic cigarette smoking is a significant risk factor for HPV transmission and the development of cytological atypia, which may progress to precancerous and cancerous lesions of the mouth. These cytologically atypical changes were found to increase dramatically in younger, chronic cigarette smokers. Oral exfoliative cytology is a non-invasive, simple, and inexpensive technique; therefore, it is ideally suited for screening at-risk populations. Tobacco smokers should, in our opinion, endure annual screenings to detect early changes for appropriate interventions.

## References

[1] D'Souza G, Kreimer AR, Viscidi R (2007). Case-control study of human papillomavirus and oropharyngeal cancer. N Engl J Med.

[2] Mousa M, Al-Amri SS, Degnah AA (2019). Prevalence of human papillomavirus in Jeddah, Saudi Arabia. Ann Saudi Med.

[3] Chaturvedi AK, Anderson WF, Lortet-Tieulent J (2013). Worldwide trends in incidence rates for oral cavity and oropharyngeal cancers. J Clin Oncol.

[4] Burd EM (2003). Human papillomavirus and cervical cancer. Clin Microbiol Rev.

[5] Carrillo-Beltrán D, Osorio JC, Blanco R, Oliva C, Boccardo E, Aguayo F (2023). Interaction between cigarette smoke and human papillomavirus 16 E6/E7 oncoproteins to induce SOD2 expression and DNA damage in head and neck cancer. Int J Mol Sci.

[6] Huang H, Zhang B, Chen W (2012). Human papillomavirus infection and prognostic predictors in patients with oropharyngeal squamous cell carcinoma. Asian Pac J Cancer Prev.

[7] Gillison ML, Koch WM, Capone RB (2000). Evidence for a causal association between human papillomavirus and a subset of head and neck cancers. J Natl Cancer Inst.

[8] Kreimer AR, Clifford GM, Boyle P, Franceschi S (2005). Human papillomavirus types in head and neck squamous cell carcinomas worldwide: a systematic review. Cancer Epidemiol Biomarkers Prev.

[9] Herrero R, Castellsagué X, Pawlita M (2003). Human papillomavirus and oral cancer: the International Agency for Research on Cancer multicenter study. J Natl Cancer Inst.

[10] Schabath MB, Villa LL, Lin HY (2014). A prospective analysis of smoking and human papillomavirus infection among men in the HPV in Men Study. Int J Cancer.

[11] Ang KK, Harris J, Wheeler R (2010). Human papillomavirus and survival of patients with oropharyngeal cancer. N Engl J Med.

[12] Ha PK, Califano JA (2004). The role of human papillomavirus in oral carcinogenesis. Crit Rev Oral Biol Med.

[13] Bancroft JD, Gamble M (2008). Theory and Practice of Histological Techniques, 6th Edition. Theory and Practice of Histological Techniques, 6th Edition.

[14] Ahmed HG, Edris AM, Mohmed EA, Hussein MO (2009). Value of centrifugated liquid-based cytology by Papanicolaou and May-Grünwald in oral epithelial cells. Rare Tumors.

[15] Hobbs CG, Sterne JA, Bailey M, Heyderman RS, Birchall MA, Thomas SJ (2006). Human papillomavirus and head and neck cancer: a systematic review and meta-analysis. Clin Otolaryngol.

[16] Mehanna H, Beech T, Nicholson T, El-Hariry I, McConkey C, Paleri V, Roberts S (2013). Prevalence of human papillomavirus in oropharyngeal and nonoropharyngeal head and neck cancer--systematic review and meta-analysis of trends by time and region. Head Neck.

[17] Schabath MB, Villa LL, Lazcano-Ponce E, Salmerón J, Quiterio M, Giuliano AR (2012). Smoking and human papillomavirus (HPV) infection in the HPV in Men (HIM) study. Cancer Epidemiol Biomarkers Prev.

[18] Hong YR, Mainous AG 3rd (2021). Electronic cigarette use and oral human papillomavirus infection among US adult population: analysis of 2013-2016 NHANES. J Gen Intern Med.

[19] Ahmed HG, Idris AM, Ibrahim SO (2003). Study of oral epithelial atypia among Sudanese tobacco users by exfoliative cytology. Anticancer Res.

